# Microbes and Viruses Are Bugging the Gut in Celiac Disease. Are They Friends or Foes?

**DOI:** 10.3389/fmicb.2017.01392

**Published:** 2017-08-02

**Authors:** Aaron Lerner, Marina Arleevskaya, Andreas Schmiedl, Torsten Matthias

**Affiliations:** ^1^The Ruth and Bruce Rappaport Faculty of Medicine, Technion Israel Institute of Technology Haifa, Israel; ^2^Department of Research, AESKU.KIPP Institute Wendelsheim, Germany; ^3^Central Research Laboratory, Kazan State Medical Academy Kazan Kazan, Russia

**Keywords:** celiac disease, bacteria, viruses, gut, microbiome, environmental inducer, environmental protectors

## Abstract

The links between microorganisms/viruses and autoimmunity are complex and multidirectional. A huge number of studies demonstrated the triggering impact of microbes and viruses as the major environmental factors on the autoimmune and inflammatory diseases. However, growing evidences suggest that infectious agents can also play a protective role or even abrogate these processes. This protective crosstalk between microbes/viruses and us might represent a mutual beneficial equilibrium relationship between two cohabiting ecosystems. The protective pathways might involve post-translational modification of proteins, decreased intestinal permeability, Th1 to Th2 immune shift, induction of apoptosis, auto-aggressive cells relocation from the target organ, immunosuppressive extracellular vesicles and down regulation of auto-reactive cells by the microbial derived proteins. Our analysis demonstrates that the interaction of the microorganisms/viruses and celiac disease (CD) is always a set of multidirectional processes. A deeper inquiry into the CD interplay with Herpes viruses and *Helicobacter pylori* demonstrates that the role of these infections, suggested to be potential CD protectors, is not as controversial as for the other infectious agents. The outcome of these interactions might be due to a balance between these multidirectional processes.

## Introduction

### Infection and autoimmunity

The relationship between infections and autoimmunity is complex. Microbial and viral infections might act as environmental triggers inducing or propagating autoimmune and inflammatory processes, resulting in symptomatic presentation of a disease in genetically high risk individuals (Lerner, 2015; Arleevskaya et al., 2017). An autoimmune disease onset following an infectious agent exposure has been well-documented (Pordeus et al., 2008; Bogdanos et al., 2015; Sakkas and Bogdanos, 2016). At least, for CD, the following infections were suggested to be associated with the disease: viruses: enterovirus, Epstein-Barr virus (EBV), Cytomegalovirus (CMV), hepatitis C virus (HCV), hepatitis B virus (HBV), and rotavirus, microbes: Bacteroides species, *Campylobacter jejuni*, Pneumococcus, *Mycobacterium tuberculosis*, and *Helicobacter pylori* (Lerner, 2015). However, recent serological evidence suggests the opposite outcome, which is the protection against autoimmune conditions following bacterial/viral exposure (Christen and von Herrath, 2005). At least the suggestive protector agents for CD were CMV, EBV, Rubella, and Herpes simplex type 1 virus (HSV1) when compared to healthy people (Plot and Amital, 2009; Jansen et al., 2016). According to the “hygiene hypothesis,” the excessively sterile environment leads to the enhanced incidence of autoimmune disorders, asthma, and allergies, thus, associating surge of CD incidence with decreased infectious environment (Lerner et al., 2015b,e; Bloomfield et al., 2016).

### Celiac disease

Celiac disease is a life-long autoimmune disease (Lerner et al., 1996) mainly of the proximal intestine, affecting genetically predisposed individuals. Gluten, the storage protein of wheat, is the environmental inducer of the disease in addition to other structurally related molecules found in barley, rye, and oat (Lerner, 2014). Many environmental factors were suggested to induce or enhance the disease: multiple infections (Lerner and Reif, 2015), early infections (Myléus et al., 2012), early gastrointestinal infections (Beyerlein et al., 2017), lack of breast feeding (Lerner and Matthias, 2016c), time and amount of gluten consumption (Chmielewska et al., 2015), microbiome/dysbiome repertoire (Lerner et al., 2015a; Lerner and Matthias, 2017a,b), mode of delivery (Decker et al., 2011), early vaccination (Kemppainen et al., 2017) or early consumption of antibiotics (Canova et al., 2014) and geo-epidemiological influences (Lerner, 1994, 2015; Reif and Lerner, 2004b; Lerner and Matthias, 2015a). The abnormal immune response is directed, in particular, against tissue transglutaminase (tTG), representing the autoantigen, (Reif and Lerner, 2004a; Lerner et al., 2015c) and the two main autoantibodies, anti-endomysium and anti-tTG antibodies, are the most prevalent serological markers used to screen for the condition (Shamir et al., 2002; Lerner and Matthias, 2015d). Recently, the list of CD serological markers was expanded by two additional autoantibodies: anti-deamidated gliadin peptide and anti- tTG neo-epitope antibodies, found to be reliable for CD diagnosis (Rozenberg et al., 2012; Lerner and Blank, 2014; Lerner et al., 2015e). As yet, HLA-DQ2 and HLA-DQ8 are known predisposing genetic factors. The sequential events in disease progression were unraveled in the last years and gave rise to multiple future therapeutic strategies (Lerner, 2010). Notably, its epidemiological, incidental, and clinical presentation are changing continuously, and new clinical pictures are reported and expand the abundance of clinical variance of the disease (Lerner et al., 2015d). In fact, age of disease onset increases and the traditional enteric presentation is more and more replaced by extraintestinal manifestations. Skin (Lerner et al., 2015d), endocrine (Lerner and Matthias, 2016d; Lerner et al., 2017), hepatic (Anania et al., 2015), metabolic (Eliyah Livshits et al., 2017), skeletal (Lerner and Matthias, 2016a), rheumatic (Lerner and Matthias, 2015b), geriatric (Lerner and Matthias, 2015c), hematological (Branski et al., 1992), neurological (Zelnik et al., 2004; Lerner et al., 2012), gynecological and infertility (Mårild et al., 2012; Casella et al., 2016), oral and dental (Cantekin et al., 2015), hypercoagulability (Lerner and Blank, 2014), cardiac (Lerner et al., 2015d), and behavioral abnormalities (Zelnik et al., 2004) are often described. Those epidemiological and clinical changes can explain why the disease is diagnosed during the whole human life-span including in the elderly (Lerner and Matthias, 2015c). There is no doubt that in the last decades its incidence is constantly increasing, ranging between 1 and 3% nowadays (Lerner, 2014; Lerner et al., 2015b). The present review will concentrate, expand and update on the multiple faces of the inductive/protective roles that infectious agents might play in CD pathogenesis. This aspect is further interesting since pathogens are the major drivers of human selective genetic adaptation during evolution (Vatsiou et al., 2016), and the question of microbes that are bugging the celiac patient “are they friends or foes?” is the subject of the current review.

### Infections and CD

It should be clarified that, although the trigger role of microorganisms and viruses in the CD development was undoubtedly traced in numerous investigations, it substantially differs from other immune pathogenesis like rheumatoid arthritis (as a classic model of an autoimmune disease; Arleevskaya et al., 2016; Kemppainen et al., 2017).

The induction of rheumatoid arthritis most likely occurs under the influence of the burden of many trivial infections, influencing the patient's immune system due to the frequent and prolonged infectious episodes (Arleevskaya et al., 2014). Individuals at CD risk apparently do not have such features in their mucosal immunity, nor significant defects in systemic anti-infective protection, impacting infection susceptibility. Since all the CD studies are focused only on the disease link with various gastrointestinal infections, such association is different from what was shown in rheumatoid arthritis (Riddle et al., 2012).

**Figure 1:**
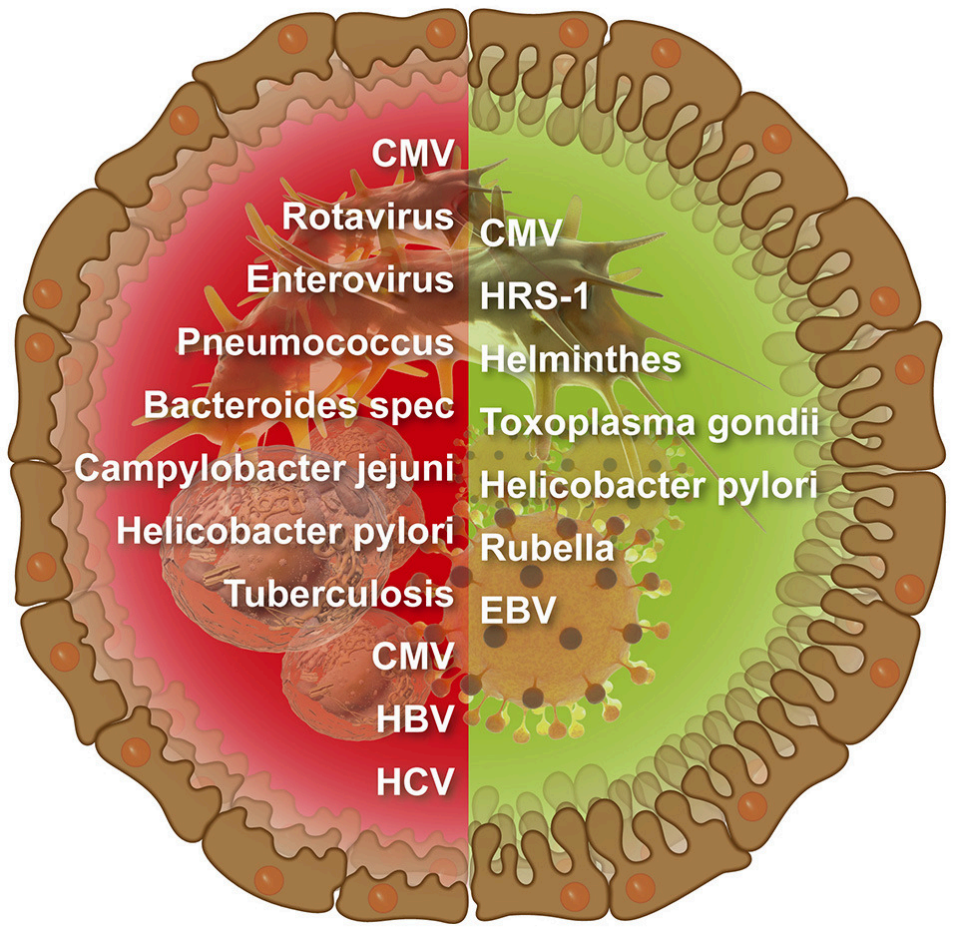
Infectious agents that were suggested to induce **(left side)** or protect against **(right side)** intestinal celiac disease.

The number of infectious agents related to CD is continuously increasing (Figure 1). Examples for viruses enterovirus, EBV, Cytomegalovirus (CMV), HCV, HBV, and rotavirus And for microbes *Bacteroides* species, *C. jejuni, Pneumococcus, M. tuberculosis*, and *H. pylori* (Lerner, 2015). However, links between CD and infections were more associative and less causative, thus, far from being elucidated. Moreover, the mutually exclusive hypotheses about the provocative and protective role of a particular microorganism/virus in CD pathogenesis were suggested and discussed in various publications.

It appears that the essential condition for the CD induction is a gastrointestinal infection (Kemppainen et al., 2017). Apparently, the other major condition is an early childhood—namely immature gastrointestinal tract, immature immune system, and gastrointestinal microbiome at the early phase of formation. The beginning of gut colonization by microorganisms set the stage for the cross talks between the epithelium, enteric lymphoid tissue, and microflora, all together establishing the intestinal barrier and as a consequence, a strictly dosed delivery of macromolecules into the internal environment and shaping of mucosal tolerance to food antigens and normal flora (Makarova et al., 2014).

So, in the vulnerable infant period any gastrointestinal infection, even a transitory one, is potentially able to disturb these processes of gut microbiome maturation and the establishment of local immunity and immune tolerance, including that against food and microbial antigens. Apparently, deleterious coincidence of these circumstances leads to an error in the negative selection of gluten-reactive lymphocyte clones.

Thus, the community of microorganisms, being extremely vulnerable during the ripening period, appears to be an inert system in adults. For example, in adults it needs no more than 30–60 days to restore gut microflora after the exposure to antibiotics (Spanhaak et al., 1998; Tannock et al., 2000). At the same time, a weekly clindamycin treatment of a newborn reduces Bacteroides diversity for the next 2 years (Jernberg et al., 2007). The same infections, capable of altering the fate of a sick infant, are merely a light ripples on the ocean surface for an adult microbiome.

In conclusion, there is an undoubted link between CD development and microorganisms, and this link looks to be rather specific. Gastrointestinal infections in predisposed infants with an immature gastrointestinal tract and immune system might shape gut microbiome in the immature and therefore labile circumstances. Such an unfortunate combination could trigger early CD development or becomes a ticking time bomb, represented by the structural features of the gut microbiome and persistence of gluten–reactive lymphocyte clones with latent basal cell proliferation without overt disruptive inflammatory activation.

## Gut microbiome signature in celiac disease

Gut microbiome analysis in the healthy adult human populations revealed about 1150 bacterial species, the majority (50–75%) being represented by Firmicutes, and then Bacteroidetes (10–50%), Actinobacteria (1–10%), with fewer than 1% being Proteobacteria (Manichanh et al., 2012). Apparently, the HLA system, to a certain extent, shapes microbiome structure. Besides, polymorphisms of some other non-HLA genes were found to correlate with a certain microbiome structure (Spor et al., 2011). Interestingly, in addition to the HLA system, microbiome composition may be due to CD-associated polymorphisms of defensin, some molecules of Toll-like receptor signaling pathways and vitamin D receptor genes (San-Pedro et al., 2005; Fernandez-Jimenez et al., 2010; Wang et al., 2016). Whole genome study of 93 individuals and 16S rRNA gene pyrosequencing of their body microflora revealed 83 alliances between genetic variance in host sequence and plethora of specific microbial taxa (Blekhman et al., 2015). In particular, the links with CD-associated host genes were revealed. In addition to the host genes related to immunity, a link was found between the microbiome composition and SNP of the genes not related to immunity. For example, the authors revealed an interesting correlation between the abundance of Bifidobacterium in the gastrointestinal tract and host genetic variation in LCT gene, encoding the lactase enzyme hydrolyzing dietary lactose. This gene SNPs are known to be associated with lactose intolerance, which is frequently associated with celiac disease (Ojetti et al., 2005). Bifidobacterium is able to metabolize lactose, and there are some strains preferring lactose instead of glucose. The authors suggested that the problems with individual's consumption of milk products might impact the richness of Bifidobacterium in the gastrointestinal tract.

The results of gut microbiome structure investigations in the infants at CD risk as well as in the therapy-naïve patients at the disease onset are somewhat contradictory. Besides the bulk of the results was obtained by study of feces, while the principal for CD microbe community in the small intestine boundary layers has its own peculiarities, although in a certain extent it is associated with fecal microbes. However, a certain tendency can be traced. A single and rather limited study of mucosa-associated microbiota in the proximal gut—using enteric samples from 45 children with CD and 18 clinical controls born during the “Swedish CD epidemic”—demonstrated only marginally differences between the groups. Enrichment with Clostridium, Prevotella, and Actinomyces was revealed in the most of the CD samples (Ou et al., 2009). Feces studies demonstrated, that infants genetically predisposed to CD, had significantly higher abundance of Firmicutes (Clostridium) and Proteobacteria (Escherichia/Shigella) and desreased proportions of Actinobacteria (Bifidobacterium) and Bacteroidetes compared to low-risk infants (Sellitto et al., 2012; Olivares et al., 2015). In a proof of concept study, Sellitto and co-workers have traced the longitudinal changes in the microbial populations colonizing from birth to 24 months in 30 genetically predisposed infants. They demonstrated that even at 2 years of age their microbiota do not resemble that of adults, while in not at-risk infants the maturation was complete at 1 year of age. The group was divided into an early and a late gluten exposure groups (17 and 13 infants, respectively). The authors showed that genetically susceptible infants may benefit from delayed gluten exposure not before 12 months of age. A hypothesis was forwarded that lack of maturity of the enteric microbiota faced with early gluten consumption can induce or accelerate the autoimmunogenetic process. Not less interesting was the infant's metabolome. When solid food was introduced at 6 months of age, succinate, acetate, propionate, and butyrate accumulated in their stools. However, by 2 years of age, butyrate, and acetate were the dominant short-chain fatty acids (SCFA). Since bacteroidetes are associated most strongly with propionate, while Firmicuters are negatively correlated to SCFAs production (Koenig et al., 2011), Sellitto's group envisioned that the high Firmicutes and low Bactriodetes abundance in CD infants results in down production of those protective SCFAs, thereby abrogating enteric health and predisposing them to autoimmune diseases. The decrease in *Lactobacillus* spp. associated with lower lactate production, observed in between 6 and 12 months of age, accompanied by decreased SCFAs' feces repertoire, during a vulnerable time of mucosal immune maturation and microbiome compositional changes might lead to loss of tolerance to non-self-antigens like gluten in those CD genetically predisposed infants (Sellitto et al., 2012). Once again it points to the important relations connecting nutrition to microbiome composition and diversity, its metabolome and the local maturation and functioning of the immune system.

From this perspective, it is interesting to compare the composition of the gut flora in infants at CD risk and premature babies with a priori immature gut. Arboleya and coauthors compared the gradual establishment of the intestinal microbiome in very-low birth-weight preterm infants with that of healthy full-term, vaginally born, breast-fed neonates using 16S rRNA gene profiling and quantitative PCR for the various microbial taxa. It was demonstrated that preterm neonates sheltered a higher relative proportion of Firmicutes at 2 days of age, and of Proteobacteria in the later sampling times, compared to control babies. Prematurity reflected reduced levels of Bacteroidetes at day 2 and as well as in later sampling times together with Actinobacteria (Arboleya et al., 2016). In addition, very-low birth-weight preterm infants frequently displayed a lag in establishing an adult microbiota compared to full-term children (Weng and Walker, 2013). So, the parallels to the peculiarities of the gut flora in CD prone individuals seem to be obvious. Combining the results of Arboleya et al. and Sellitto et al. about the enteric mucosal immaturity and the unbalanced microbiome found early in life and in CD high-risk infants, it can be suggested that by ingesting gluten peptides, the disease will progress in these individuals, unlike in non-CD high-risk premature infants, that will never develop CD. The features of CD gut microbiome, incorporated in early childhood seem to persist in the adulthood even despite gluten withdrawal (Nistal et al., 2012; Wacklin et al., 2014).

Caminero and co-workers demonstrated that gluten amounts in feces of healthy volunteers, CD patients and individuals under risk receiving gluten-free or normal diet depended on gluten intake. The greatest amount of gluten was found in fecal samples from healthy volunteers being on normal diet, with a significant decrease in the untreated CD patients and the individuals under risk. It is noteworthy that in all the groups fecal peptidase activity against the gluten-derived peptide 33-mer inversely correlated with gluten amount in the samples (Caminero et al., 2015). It looks like the increased functional proteolytic activity of gut microflora in CD patients can affect gluten excretion. The same research group isolated 144 strains belonging to 35 microbial species that might be involved in gluten digestion in the human intestine. Most of the strains were part of the phyla Firmicutes and Actinobacteria, mainly from the genera Lactobacillus, Streptococcus, Staphylococcus, Clostridium and Bifidobacterium (Caminero et al., 2014). Ninety-four of these strains were capable to metabolize gluten, 61 of them showed an extracellular proteolytic activity of gluten proteins, and several strains showed a peptidase activity toward the “supra-molecule” 33-mer peptide, the luminal immunogenic molecule in CD patients (Caminero et al., 2014). At the end of the day, there is a certain difference in gluten proteolysis by various bacteria, and the immunogenicity of the generated peptide fragments might be different. It should be noted that these studies were carried out in cultures, in which the glutenase activity of aggregate microbial community as a whole (biofilm) in the gut boundary layer might significantly differ from the isolated activity of the individual members of this community.

It is known that the microbiome impact gastrointestinal and systemic functions by its metabolome, the most studied one being the short chain fatty acids (SCFA). Most recently, the topic of nutrition, microbiome, and SCFA associations in CD was updated (Lerner et al., 2016). Multiple beneficial effects to the host were attributed to them. Changes in microbiota and their SCFA production is clearly related to the pathogenesis of CD. Interestingly, peculiar dysbiosis and significant changes in stool SCFA profile were described in several autoimmune diseases, one of those is in Behcet's disease where decreased butyrate production was suggested to play a role in its pathogenesis (Consolandi et al., 2015).

Taken together, the microbiota/dysbiota disbalance may present a risk factor for CD either directly by influencing the mucosal immune responses or by intensifying inflammatory responses to gluten. In contrary, several microbial species are capable to break down gliadin and perhaps therefore decrease the immunopathogenicity of consumed gliadin (Sjöberg et al., 2013; Carding et al., 2015; Lerner and Matthias, 2015a; Rostami-Nejad et al., 2015).

## Coexistence of certain infections and CD: a critical look at the issue

It is of interest how CD affected individuals survived in the presence of harmful conditions (increased gluten content and toxicity in wheat, increased gluten consumption worldwide; Lerner et al., 2017) and despite them, thrived and expanded? (Lerner, 2011; Lerner et al., 2015b). Despite being underprivileged with nutritional deficiencies, failure to thrive and high morbidity and mortality, a substantial increase in the disease incidence is observed in the last decades. There are several theories explaining this paradox. One is positive evolutionary selection, in which the celiac patient accumulates protective genes (Lerner, 2011). The other is due to some pathogens which are the major drivers of human selective genetic adaptation (Vatsiou et al., 2016) that could have been beneficial environmental factors, protecting the CD populations. Indeed, in contrast to the observations that infections may induce CD, some infection agents were assumed to have a protective impact (Christen and von Herrath, 2005; Gaisford and Cooke, 2009; Kivity et al., 2009; Plot et al., 2009).

This assumption was based in particular on a lower incidence of the serum antibodies against Cytomegalovirus (CMV), EBV, and Rubella in CD patients when compared to healthy people (Plot and Amital, 2009). Another argument for the protective effect is the inverse correlation between serum anti-CMV, -EBV, and/or -Herpes simplex type 1 virus (HSV1) IgG levels and anti-tTG antibodies (Jansen et al., 2016).

### Viruses

It should be specified that Jansen and coauthors determined the antiviral antibody levels in the sera samples of 6-year old children (Jansen et al., 2016). CMV single infection and combined CMV, EBV, and/or herpes simplex virus type 1 infection antibodies were inversely associated with strongly tTg-IgA positivity. The authors suggested that the serological profile may indicate a protective effect of herpesvirus infections in the pathogenesis of celiac disease autoimmunity. It is accepted that by this age IgG and IgM production is close to that of the adults. However, the immune system is still immature and its reaction is not fully functional at this age even in healthy children, whereas the immune system of CD prone children, at least, the local immune defense, is somewhat delayed in the maturation. Thus, Jansen et al. suggested explanation should be taken with a grain of salt.

In Plot's publication, the age profile of the studied cohorts was not presented (Plot and Amital, 2009). There are some curious details in this publication which need to be interpreted. First, while the prevalence of the anti-EBV capsid antigen and anti-EBV nuclear antigen IgG in the CD patients was significantly lower than that in the controls, the prevalence of the anti-EBV early antigen IgG was comparable in the two groups and the prevalence of anti-EBV capsid antigen IgM, though unreliably, was more than twice higher in the CD group. In general, the anti-EBV capsid antigen IgMs are known to be produced during the acute phase (the first days—6 months from the onset of the disease) or during acute exacerbation of chronic EBV infection (http://www.tiensmed.ru/news/epstein-barr-bc1.html#nov1). In addition, if the anti-EBV early antigen IgG is not concurrently revealed, it may indicate incubation period or the very beginning of the infection (up to 1 week of symptoms). So, either in the CD patients this generally latent infection is apt to more frequent exacerbations, or, given the reduced frequency of the studied IgG antiviral antibodies, the existence of some features of the antiviral antibody production in CD can be assumed. Secondly, revealed by PCR, CMV DNA presence in the samples is not always accompanied by the presence of serum specific antibodies. This situation is typical in particular for the infants with immature immune system (Dong et al., 2004, 2005).

A number of publications on the HBV vaccine non-responsiveness in CD might attest for possible unique features of the antiviral antibody response formation (Noh et al., 2003; Park et al., 2007; Urganci and Kalyoncu, 2013), further criticizing the assumption that decrease antibodies activities in CD patients represent a protective effect.

As for the inverse correlation of the antiviral and anti-tTG antibodies demonstrated by Jansen et al. (2016), similar patterns are not uncommon in autoimmune diseases, and this fact by no means demonstrates a lesser exposure to the infections. For example, high levels of autoantibodies against double-stranded DNA, reflecting the activity and severity of systemic lupus erythematosus, are quite often combined with lower levels of antibodies to particular bacterial DNA (Pisetsky and Drayton, 1997). The inverse correlation of those indexes is usually explained by a distorted immune humoral response.

Additionally, it should be specified that, in general, in Herpesvirus infections and viral hepatitis the specific antibodies are not the principal players in the antiviral defense but are accepted to be the reliable serological markers for an infection (Grinde, 2013). It is advisable to note that caution should be used for the interpretation of the protective role of these infections in CD, relying only on data on the incidence of the corresponding antibodies.

As for Rubella infection, the specific antibodies are the major antiviral response players. However, the incidence of CD in children vaccinated with inactivated rubella virus as part of polio vaccine was close to that in the unvaccinated children (Myléus et al., 2012). Thus, a reduced incidence of the anti-rubella IgG antibodies demonstrated by Plot and co-workers can mean an equally probable lower exposure of CD patients to Rubella and the above-discussed features of the specific IgG antibody production. Moreover, since the antiviral immune response is always multi-componental, the disturbed antibody formation, although being a weak link of an antiviral defense, does not necessarily entail an increased susceptibility to Rubella.

The data which might testify for the direct and reverse links of CD to Herpes virus infection are summarized in Table 1. The analysis of the data demonstrates that the links of CD and herpes infections are multi-directional. On one hand there are some peculiarities, which can promote the viral infecting: (1) CD-associated DQA1^*^0501/DQB1^*^0201 genotype, which is also due to the imperfect response against Herpes viral infection; (2) the typical CD immature gastro—intestinal tract and the delayed process of microbiome maturation, which might be risk factors for the virus infecting; (3) the typical CD mucosal overexpression of epidermal growth factor receptors, by which Herpes viruses enter the cells; (4) increased expression of IL-33, suppressing local antiviral immunity in CD patients. On the other hand, the increased levels of several humoral factors with the antiviral activity; increased expression of some cytokines, which promote mucosa maturation and thus increase its' resistance to the viruses; as well as some potential features of CD microbiome that might indicate a backward link between CD and Herpes viral infection. The protective effect of the infection on atopic manifestations was demonstrated in the case of the early (infancy or early childhood) EBV exposure, while the later infection predisposes to the atopic disease (Nilsson et al., 2005, 2009). So, if to extrapolate the data on the links of herpes and atopic diseases to CD, it is likely that the early (infancy or early childhood) EBV exposure might play a protective role, while the later infection might trigger CD or have no impact on it at all.

**Table 1:** Direct (⇑⇑) and reverse (⇑⇓) links of CD and Herpes virus infections.

**⇑⇑**	**⇑⇓**
Markedly impaired binding and presentation of some herpes antigens to the TCRs in CD-associated DQA1^*^0501/DQB1^*^0201 carriers (Koelle et al., 1997; Reichstetter et al., 1999) might be due to the imperfect antiviral immune response as well as to the peculiarities of antibody production.	–
Increased HSV2, CMV and EBV DNA levels in the stool samples were observed among premature neonates with intrauterine growth restriction compared with those infants born appropriate for gestational age (Naing et al., 2013). The immature gastro—intestinal tract and the delayed process of microbiome maturation might be the typical signs of CD (Sellitto et al., 2012), that might be risk factors for the virus infecting in infancy as well as for the higher rates of the clinical manifestations of the infection.	Virus-shaped cytokine levels might to some extent promote the maturation of the local immune system and intestinal tissues, lagging behind in the CD-prone individuals: IL-6 promotes enterocyte differentiation and inhibits enterocyte apoptosis, TNF-alpha promotes intestinal growth (Rollwagen et al., 1998; Maheshwari, 2004), IFN-gamma increases macromolecular transport in the immature gut particularly across Peyer's patches. This Peyer's patch-targeted effect can be important for setting mucosal immune responses against dietary antigens early in life and aiding their immune exclusion (Sütas et al., 1997).
Herpes viruses were revealed in the inflamed gastrointestinal tract mucosa, but never in the endoscopically healthy tissue (Ramanathan et al., 2000; Roblin et al., 2011). It is unclear whether the inflamed mucosa is a consequence of the viral infection or the inflamed tissues “draw” viruses, due to the expressing of the corresponding receptors. Epidermal growth factor receptors, by which Herpes viruses enter the cells are overexpressed in CD gut mucosa, that being due to gliadin stimulatory effect (Barone et al., 2007; Juuti-Uusitalo et al., 2009).	In CD the level of various humoral factors with a pronounced diverse direct and indirect antiviral activity in the inflamed intestinal tissues are increased (defensins, IFN-gamma, IFN-alpha, TNF-alpha, IL-6, IL-15 the latter being necessary for the development and function of NK/NKT cells and maintenance of naive and memory CD8(+) T cells; (Forsberg et al., 2004; Hazrati et al., 2006; Di Sabatino et al., 2007; Brottveit et al., 2013; Meresse et al., 2015)), that might have a protective effect on the infection.
Microbiome features might impact antiviral immunity via stimulation of IL-33 (alarmin) released by mucosal epithelium, which suppresses local antiviral immunity by blocking the migration of effector T cells to mucosa, thereby inhibiting the production of IFN-γ, a critical cytokine for antiviral defense, at local infection sites (Oh et al., 2016). Serum levels and intestinal tissue expression of IL-33 and its receptor in CD patients were found to be increased (López-Casado et al., 2015).	Pre-treatment of HeLa monolayer with inactivated *Staphylococcus aureus* cells before HSV infection increases expression of TNF-a, IL-6, and IL-8 genes, that being due to the protection from the occurrence of virus mediated cytopathic effect and to decrease of viral multiplication rate (Bleotu et al., 2015). Vaginal Lactobacillus strains neutralize lactic acid and thus, acidic pH values needed for the viral replication, as well as to macrophage activation (Conti et al., 2009; Khani et al., 2012). Microbiota might impact the antiviral defense via regulation of the natural killer T cells at the frontiers of the mucosal immune system (Zeissig and Blumberg, 2014). So, certain shifts in the structure of the microbiome mighty inhibit viral infection.

### *Helicobacter pylori* (Hp)

The permanent interest in CD and Hp infection coexistence is quite natural, due to the gut-stomach axis (Lerner and Matthias, 2016b). The infectious inflammatory process directly in the gastrointestinal tract—CD epicenter, which might shape the local immune system and microbiota, might obviously play a role in CD pathogenesis. In addition, both CD and Hp infection in a number of cases are associated with the diffuse lymphocytic gastroenteropathy (Lynch et al., 1995; Broide et al., 2007; Pai, 2014). However, diffuse lymphocytic gastroenteropathy is far from being obligatory attributed only to both entities (Wu and Hamilton, 1999; Nielsen et al., 2014). Besides, lymphocytic gastritis and a subsequently villous atrophy are accepted to be a non-specific manifestation of many pathological conditions in the gastrointestinal tract, due to a wide variety of infectious, immunologic or any inflammatory stimuli raising intraepithelial lymphocyte numbers. Lymphocytic duodenitis and increased intraepithelial lymphocytosis are known to be associated with diseases that are completely different in their pathogenesis, such as autoimmune disorders like CD (Broide et al., 2007; Rostami et al., 2010), tropical sprue, food protein intolerance, Hp-induced duodenitis, peptic duodenitis, parasitic, and viral infections, intestinal lymphoma (Chang et al., 2005; Brown et al., 2006; Pallav et al., 2012; Rosinach et al., 2012; Shmidt et al., 2014) drugs' induced duodenitis (non-steroidal anti-inflammatory drug; Shmidt et al., 2014) and small-intestine bacterial overgrowth (Lappinga et al., 2010). The differential diagnosis of lymphocytic gastritis is not less restricted. CD and HP are not the only ones. Various non-HP infections, inflammatory conditions and several non-celiac autoimmune diseases were described (Broide et al., 2007; Polydorides, 2014).

The interest in the CD—HP infection link is fueled by the well-known data, indicating that childhood infection with HP could protect against the development of Crohn's disease, severe gastric-reflux disease, Barrett's esophagus and esophagus adenocarcinoma (Chen and Blaser, 2008). Yet, as for the protective role of Hp in CD, the available data are limited and quite contradictory. The prevalence of CD among Hp-positive adults was 0.05% compared with 0.09% among Hp-negative individuals (statistically non-significant) while the prevalence of Crohn's disease among Hp–positive patients was 0.07% compared with 0.24% among Hp-negative patients (Bartels et al., 2016). Based on these data, at least in adults, the protective effect of Hp on CD is minimal, if at all existing. However, given the fact that in childhood gastrointestinal infection appears to be a more important condition for CD triggering than in adults, this conclusion is not necessarily true in the case of early exposure to Hp (Kemppainen et al., 2017). Unfortunately, we failed to find the similar data on the CD frequency in children infected with Hp early in life.

The incidences of Hp and CD worldwide vary enormously (9–100%, 0.3–3.9%, respectively; Rostami-Nejad et al., 2016). Most of the studies aimed to determine the ratio of Hp infected individuals in CD and non-CD control groups. In our opinion, this study design gives less information about the inductive/protective effect of Hp infection on the CD development as on the susceptibility of the CD patients for the infection. The results of various studies on Hp both in children and adults are contradictory (Table 2). The spread in the ratios of both Hp infected CD patients and non-CD controls in these publications might be due to the national and age-related characteristics of the studied cohorts (Eusebi et al., 2014). As for the diametrically opposed regularities of HP incidence in CD and non-CD groups, a meticulously analysis of these publications, did not lead us to any reason for the conflicting results, but to the possible differences in the poorly described clinical characteristics of the controls. In these studies, the authors examined the collections of endoscopically obtained biopsies and sera allocating the cases into CD and non-CD (control) groups. It is obvious that the persons accepted as controls underwent the relevant examination because they had any gastroenterological problems associated or not associated with HP infection.

**Table 2:** The incidence of *Helicobacter pylori* infection (Hp-positive, %) in CD patients and non-CD controls.

**Age characteristics of groups**	**Hp-positive CD patients, %**	**Hp-positive non-CD controls, %**	**References**
Adults	36%	41%	Simondi et al., 2015
	86% (82% untreated, 95% treated)	97%	Diamanti et al., 1999
	20.7% (Untreated) 32.4% (treated)	55.3%	Ciacci et al., 2000
	Frequency increased with age in groups.		
	12.5%	30%	Lasa et al., 2015
Children (age, years)	21.8% (Median 8.2 years)	23.8% (Median 8.9 years)	Aydogdu et al., 2008
	18.5% (Median6.8 years)	17.3% (Median6.8 years)	Luzza et al., 1999
	5.4% (3–12 years)	6.8% (3–12 years)	Jozefczuk et al., 2015
	30.6 (< 18 years)	33.8% (< 18 years)	Guz-Mark et al., 2014
	2.7% (Median5.7 years)	15.6% (Median7.4 years)	Nenna et al., 2012
	11.4% (1–18 years)	50% (1–18 years)	Narang et al., 2016

The analysis of the literature data which might testify the direct and reverse links of CD and Hp infection (Table 3) shows that the interaction of the two diseases represents an interweaving of differently directed processes. Perhaps the end result might depend on the balance of these processes, being deeply individual in each specific case. Important is the assumption that the direct or reverse CD/HP link may depend on the age at which the encounter with the bacterium occurred. At least, it is important for allergies–Hp links. Our attempt to test this hypothesis failed, because the literary data accumulated to the present moment are largely insufficient.

**Table 3:** Direct (⇑⇑), reverse (⇑⇓) or no (⊗) links of CD and Hp infection.

	**⇑⇑**	**⇑⇓ or ⊗**
Clinic	The cases of fresh HP infection in the CD patients and the reports of CD onset right after the carried *H. pylori* infection both in children and adults were described (Cârdei et al., 2003; Villanacci et al., 2006).	The milder CD forms were found to be more prevalent in HP-positive adults (Villanacci et al., 2006). The data, received in children are contradictory (Guz-Mark et al., 2014; Narang et al., 2016). On a gluten free diet the normalization of the duodenal mucosa was independent of presence/absence of HP both in adults and children (Bardella et al., 2007; Aydogdu et al., 2008).
Genetic	DQA1*03:01 was found to be often in both CD and HP-positive duodenal ulcer patients (Azuma et al., 1995; Mubarak et al., 2013). TNF-308 (G > A) SNP increases risk of CD and persistent HPinfection (Khan et al., 2016). The−336G CD209 allele^#^ associated with a higher HP infection severity/susceptibility might be involved in CD susceptibility in HLA-DQ2 negative patients. (Núñez et al., 2006).	DQA1^*^0201 allele associated with the high CD risk was significantly rarer in the HP-positive duodenal ulcer patients than in the HP-negative controls (Azuma et al., 1995; Lionetti et al., 2014). HLA-DRB1^*^0301 and DRB1^*^07 associated with high CD risk in some ethnic groups are involved in the of some HPproteins recognition and in subsequent activation of gastric T cells within the framework of antiinfective immune response (Bilbao et al., 2002; talová et al., 2002; Bergman et al., 2005).
Gastrointestinal functions	Hp infection leads to the abnormal gut permeability, due to increased production of pro-inflammatory cytokines (Caron et al., 2015). At that the subjects with latent CD have an abnormal permeability (Peña and Crusius, 1998; Sapone et al., 2011) The abnormal mucosal permeability increases the gluten availability for the gluten-specific lymphocyte clones in the Peyer's Plaques.	CD patients were found to have high basal and stimulated acid-forming function, while a comfortable microenvironment for HPincludeЪɪhypochlorhydria (Il'chenko et al., 1991; Savarino et al., 1999; Krums et al., 2011; Harris et al., 2013). Indeed, HPcan only survive for minutes in the stomach lumen (pH of 1–2) and must quickly migrate to the gastric epithelial surface (Schreiber et al., 2005).
Innate immune	–	Hpcolonizes the gastric mucosa by adhering to the mucous epithelial cells via the fucosylated blood group antigens H-type 1 and Leb (Magalhães and Reis, 2010). In principle, the typical for CD immaturity of the gastrointestinal tractincludes weak functions of the mucosal barrier and a lack of bacteria colonization (Forchielli and Walker, 2005). However, single publications indicated no features of the glycocalyx/mucous layer carbohydrate structures in CD (Toft-Hansen et al., 2013).
	The inflammatory cells in the Hpinfection in epithelium and lamina propria express inducible NO-synthase with excess free radicals due to the alterations and exacerbation of inflammation with impaired regeneration processes (Cherdantseva et al., 2014). The similar events occur after gluten exposure in the gastrointestinal mucosa of gluten-sensitive patients, being due to the in injury of the small-intestinal tissue (Holmgren Peterson et al., 1998; Niveloni et al., 2000).	Increased tissues concentration of nitric oxide metabolites in CD might have a protective effect against Hp (Gobert and Wilson, 2016). At the same time Hp uses diverse strategies to promote its survival. All Hpstrains encode proteins important for detoxifying reactive oxygen species and its arginase limits NO production by macrophage-, neutrophil- and epithelial cell-derived nitric oxide synthase (Salama et al., 2013). So, the passing suppression of CD- caused inflammation by Hp is quite possible. Defensins' levels are increased in CD mucosa (Vordenbäumen et al., 2010). During Hp infections, these cationic peptides with antimicrobial properties play a pivotal role in the innate immune responses and are able to eradicate the bacteria (Pero et al., 2017). It should be noted that according to some data, HP strains were resistant to these factors (Nuding et al., 2013).
	Hp induces apoptosis of gastric epithelial cells directly and via modulation TRAIL-mediated apoptosis signaling (Tsai and Hsu, 2010). This effect might contribute to epithelial apoptosis and villous atrophy—CD hallmark (Shalimar et al., 2013).	In Hp positive CD patients a significantly lower prevalence of atrophic gastritis was observed when compared with Hp negative ones (Santarelli et al., 2006). That might be due to the expression of several DNA repair proteins in the inflamed tissue accumulating damaged host DNA (Salama et al., 2013). On the other hand, Hp can gain a foothold in gastrointestinal tract only in the mild CD cases. It is in line with the data, demonstrating the higher inflammation in correlation with lower bacterial upload (Salama et al., 2013).
	Proinflammatory cytokine production in the framework of anti Hp immune reaction might be due to CD triggering (Crabtree, 1996).	Increased local levels of proinflammatory cytokines in CD might have a protective effect against the fresh Hp infection (Eiró et al., 2012; Di Sabatino et al., 2016).
	Gastroduodenal response to chronic Hp infection include IL-8secretion, that being due to neutrophil migration and activation (Crabtree, 1996). These events might trigger CD, as the cells play important role in CD pathogenesis (Lammers et al., 2015).	The increased infiltration by activated neutrophil was demonstrated in CD mucosa (Hällgren et al., 1989). That might have a protective effect while HPexposure.
Adaptive immunity	After the challenge of 4-weeks aged (infants) and 6-weeks aged (adults) mice with HP strain T-cell activation in the gastric samples was demonstrated including the pathways for pro-inflammatory molecules (nitric oxide, iNOS), this effect increased over time (Kienesberger et al., 2016).	Hp re-programs dendritic cells playing a crucial role in Hp recognition toward a tolerance-promoting phenotype; HP-exposed DCs fail to induce effector T-cell responses of the Th1 and Th17 type *in vitro* and *in vivo*; instead, they preferentially induce the expression of the Treg-specific transcription factor FoxP3, the surface marker CD25 and the anti-inflammatory cytokine IL-10 in naive T-cells (Salama et al., 2013). The role of this effect in protection from protection against allergies, asthma and inflammatory bowel diseases, was demonstrated (Oertli and Müller, 2012). Besides, at least two virulence factors are known to inhibit human T-cells. VacAvia β2 integrin reception inhibits T-cell proliferation and prevents nuclear translocation of the T-cell transcription factor NF-AT and its subsequent transactivation of T-cell-specific immune response genes. The other virulence determinant—γ-glutamyl-transpeptidase blocks proliferation of T-cells (Salama et al., 2013). As the interaction of the virulent molecules with T-cells is non-specific, a bystander gluten-specific T-cell repression is quite possible. It's a purely speculativehypothesis. No one research was published on this issue.
Microbiome	Lactobacillus species (*Lactobacillus johnsonii*, Lactobacillus murinus, *Lactobacillus reuteri*) were able to inhibit Hpgrowth *in vitro* (Zaman et al., 2014; Delgado et al., 2015). *Streptococcus mitis*, a commensal microorganism of the human stomach, was found to inhibit Hpgrowth and to drive its conversion from a spiral to a coccoidal form (Khosravi et al., 2014). Just the same Lactobacillus and Streptococcus species appeared to be ability to degrade and remove gluten derivatives (Fernandez-Feo et al., 2013; Duar et al., 2015). The impact of these coincidences on both CD and Hp infection remain unclear because (1) the studies of activities were carried using cultivated and then isolated microbes, whereas the glutenase activity of aggregate microbial community as a whole (biofilm) in gut boundary layer may significantly differ from the isolated activity of the individual members of this community; (2) the immunogenicity of the generated peptide fragments might be different that might be due to the opposite effect on CD.	

*#CD209 is a dendritic and macrophage surface molecule involved in pathogen recognition and immune activation, Hp infection in particular (Bergman et al., 2006; Núñez et al., 2006). It was found to be overexpressed in the Hp infected gastric epithelial cells and to mediate Th1 differentiation, which may be involved in gastric mucosal injury (Wu et al., 2014)*.

## Potential mechanisms for beneficial bugs' effects in celiac gut

Multiple potential pathophysiological avenues were suggested to understand the microbial-gut cross-talks in CD.

### Post-translational modification of protein (PTMP) from non-self- to self-proteins

Endogenous and microbial enzymes are capable to generate intestinal enzymatic neo-antigens via PTMP. The modifications taking place in the intestinal lumen include peptides crosslinking, de/amination/deamidation by the transglutaminases, de/phosphorylation, a/deacetylation, de/tyrosination, and many other enzymatic modifications exist (Lerner et al., 2016). Related to the present topic, the human endogenous intestinal enzymes, tTG and its family member, the exogenous microbial transglutaminases, induce multiple neo-epitopes on the TG-gliadin cross-linked complex resulting in the formation of antibodies against the complexes in CD. CD is a classical disease where luminal PTMP is driving the disease. It seems logical that a microbial agent might modify non-self-peptide to self-one—reducing its immunogenicity. Additionally, in CD, some microbe strains might modify gluten in the lumen, thus preventing or aggravating the inflammatory cascade and the intestinal damage progression via PTMP (Caminero et al., 2016).

### Horizontal gene transfer in the human gut lumen

Given the extensive influence of the microbiota on human health, the gut-microbiome integrity is of prime importance for host health and survival. In this regards, our bodies' “second genome” cohabit with the human one to form a stable equilibrium for the two kingdom's long term survival. As opposed to long-term evolutionary events, newer genetic manipulations with microorganisms, plants, animals, or nutrients, applying new food technologies and/or microbial engineered delivery systems or novel mode of therapies are rapidly evolving.

Due to the close relationship and intimate cross-talks between the human and the gut's biospheres, consumption of the modified genetic cargo into the human intestinal ecosystem might occur. It was hypothesized that modern probiotic ingestion, genetically manipulated food consumption and genetically manipulated microorganism usage are potential genetic driving forces for changing the evolutionary equilibrium established during the last millions of years (Cho and Blaser, 2012). Horizontal gene exchange is the ability to transfer genetic material between contacting biological domains, including eukaryote (plants, animals, and man), prokaryote (microbes), and viruses (Aminov, 2011; Ruggiero et al., 2015). Despite not being investigated in CD, various virulent genes, the most studied one is the antibiotic resistance gene, were described to be laterally transferred. It is hypothesized that the opposite might occur. This infectious genetic cargo might include anti-inflammatory/pro-apoptotic/Treg or other immune-modulatory genes, attenuating or abrogating autoimmunity.

### Infections as tight junction closure enhancers

The tight junction protein, Zonulin, is involved in the regulation of the intestinal permeability between gut epithelial cells. Several clinical trials with Zonulin antagonist (Larazotide acetate, AT-1001, Alba Therapeutics, USA) demonstrated the promising therapeutic effect in CD (Lerner, 2010; Khaleghi et al., 2016). Larazotide acetate—an octa-peptide derived from a cholera toxin ZO, antagonizing zonulin via receptor blockade—is aimed to decrease the paracellular transport caused by gluten and thus to suppress the activation of the pathological immune cascade. In addition to the above mentioned cholera toxin derivative, many other factors produced by microorganisms can improve tight junction performance, modulating intestinal permeability. *Salmonella enterica serovar, Escherichia coli*, and *C. jejuni* modulated enteric epithelial barrier functions in chickens (Awad et al., 2012, 2014, 2015), and the probiotics *Lactobacillus casei* DN-114001 and *E. coli* strain Nissle 1917 decreased intestinal epithelium permeability in human intestinal originated cell lines (Parassol et al., 2005; Zyrek et al., 2007; Trebichavsky et al., 2010). Taken together, modulation of gut permeability by infectious agent, counteracting the breached tight junction integrity in CD, might represent a protective pathway.

### Molecular mimicry between infectious agents and self-antigens

Generally, molecular mimicry between foreign (infectious/environmental) and self-antigens is a well-described pathway of autoimmune disease induction. Recently, it was suggested that antigen mimicry between foreign and self-antigens might be due to the long-term regulation of inflammation (Pontes-de-Carvalho et al., 2013). In a cohort of African patients, infected with *Schistosoma* it was found that the parasite inhibited production of anti-nuclear antibodies (Mutapi et al., 2011). More recently, apparent effectiveness of rotavirus vaccination was found to prevent the onset of CD autoimmunity (Silvester and Leffler, 2016). This finding gives indirect evidence for the persistence of regulatory cells in the lack of stimulation of the immune system by pathogen-derived processes. The strength of the immune adjustment, however, may increase with the uninterrupted presence of the pathogen or its antigens. Thus, molecular mimicry can represent a protective mechanism of autoimmunity.

### Th2 to Th1 shift

Inflammation, given rise by microbes, viruses and especially by parasites such as helminths, can shift the Th1 pathway to Th2 one, resulting in a more immunosuppressive state where regulatory T cells might be induced or be activated (Shor et al., 2013). Recent studies have supplied such an evidence for pathogen-specific regulatory cells in *Leishmania major*, Herpes simplex virus, and Friend retrovirus (murine leukemia virus) infections (Christen and von Herrath, 2005). During the acute phase of the infection this Th2 profile counter-regulates Th1-driven autoimmune pathologies. Along the chronic stage of infection, immune-regulatory networks arise, mainly led by regulatory T cells. These cells produce IL-10 and TGFβ, which has observative effect on Th1-related autoimmune diseases, such type 1 diabetes mellitus or CD. In fact, several helminths were tried in CD patients with encouraging results (Croese et al., 2015). Treatment with helminthes or helminthes ova ameliorated the clinical pictures of several autoimmune conditions in patients as well as in animal models (Smallwood et al., 2017). A major recent contribution to the field is the helminth phosphorylcholine proved to be an immunemodulatory molecule. Most recently, tuftsin-phosphorylcholine, a novel helminth-based compound was shown to reduce pro-inflammatory cytokine production and induced anti-inflammatory cytokine expression and Treg and Breg cell expansion in mouse models of rheumatoid arthritis, lupus nephritis, and colitis (Bashi et al., 2015b, 2016; Shor et al., 2015).

It is conceivable, that the ability of helminthic parasites to attenuate host immune responses into an anti-inflammatory/regulatory phenotype is attributed to the endogenous component that the parasites secrete and/or excrete interacting with immune effector cells to regulate their function (Lund et al., 2014; Selmi, 2016).

An additional mechanism was suggested for the helminth's immunomodulation of autoimmunity, in addition to the Th1 to Th2 shift. Accelerated T and B regulatory phenotypes, decreased levels of the inflammatory cytokines like IFNγ and Il-17 or vice versa, promoting IL-4, IL-10, and TGF-β release (Bashi et al., 2015a). Since CD is a Th1 profile disease, shifting the immune pathway to Th2 profile might reduce the intestinal damage (Lerner, 2010).

### Immune activation induced cell death

Inflammation can cause a substantial hyperactivation of auto-aggressive lymphocytes, leading to activation-induced cell death and attenuate the systemic load of aggressive T cells. It seems that repeated encounter with powerful antigenic stimuli leading to restriction of an immune response is well-established in viral infections, where the primary response undergoes a major restriction after antigen elimination. EBV, HBV, and CMV infections are some of the examples. Similarly, administration of mycobacterial products, such as bacilli *Calmette-Guérin*, prevented the onset and recurrence of type 1 diabetes mellitus in NOD mice by inducing apoptosis of autoreactive T cells (Christen and von Herrath, 2005). In view of the fact that viruses are inducers of immune cells apoptosis while sparing the Treg cells (Che et al., 2015) and apoptosis is enhanced in CD (Shalimar et al., 2013), it is suggested that viruses, by abrogating immune activation, might attenuate intestinal autoimmune progression in CD.

### Infection at another location might keep auto-aggressive cells from reaching the site of autoimmune destruction

As suggested by Christen (Christen and von Herrath, 2005), an infectious inflammation elsewhere in the body might keep auto-aggressive cells from arriving into the sites of autoimmune destruction, that might be due to the abrogation of type 1 diabetes in NOD mice after LCMV infection. The authors suggested that this occurred because the “abrogative” virus grew predominantly in peripheral lymphoid organs and other sites rather than the pancreas or its islets themselves. Thus, the sites of severe inflammation might act as a filter for auto-aggressive T cells removing them from the circuit and depriving them from homing the pancreatic islets. Similar scenarios might operate where infection with B. coxsackievirus or *Salmomella typhi murium* protected against autoimmunity (Tracy et al., 2002; Raine et al., 2006).

### Immunosuppression by extracellular vesicles

Release of extracellular vesicles is a natural phenomenon of almost all cell types. They derive either from multivesicular bodies or from the cellular plasma membrane. Those vesicles contain a subset of cell derived proteins, lipids, including nucleic acids. Extracellular vesicles regulate immune responses against pathogens, as well as autoimmunity. It is suggested that these suppressive vesicles would prevent peripheral self-antigens and commonly encountered foreign antigens from causing chronic inflammation and autoimmunity. Following this lines, it is hypothesized that various infectious agents can induce those regulatory extracellular vesicles, counteracting autoimmune pathways, playing a protective anti-autoimmune role (Robbins and Morelli, 2014; Robbins et al., 2016).

### Infectious agents' secretion of anti-autoreactive T cells proteins

Infections with helminths can prevent or attenuate auto-inflammatory/immune diseases. In addition to their Th1 to Th2 shift, most recently, Helminth secreted proteins were shown to prevent autoimmunity. The excretory/secretory products of Fasciola hepatica contain immune-modulatory molecules that arbitrate protection from autoimmune diabetes via the activation and provision of a regulatory immune environment (Lund et al., 2014). Such a mechanism was not studied in CD, but might explain the new potential therapeutic strategy to treat CD with *Necator Americanus* larvae (Croese et al., 2015; Giacomin et al., 2015).

## Conclusions

The cross-talks between infections and autoimmunity are complex (Figure 1). Most of the data indicate that microbes and viruses are major environmental factors in autoimmunity induction. However, growing evidences conversely suggest that infectious agent can abrogate or protect against autoimmunity. This protective evolutionary cross-talks between microbes/viruses and us might represent a mutual beneficial equilibrium relationship between two cohabiting ecosystems. The protective pathways might involve PTMP, decreased intestinal permeability, Th1 to Th2 immune shift, induction of inflammatory immune cell apoptosis, auto-aggressive cells relocation from the target organ, immunosuppressive extracellular vesicles and anti-autoreactive cell immune-regulatory proteins.

Yet, our analysis demonstrates that the interaction of the microorganisms /viruses and CD is always a set of multi-directional processes. With a detailed consideration of possible mechanisms of CD and CMV, EBV, Herpes simplex type 1, Rubella, *H. pylori*, it can be assumed that the role of these infections suggested to be potential CD protectors infections, is not so unambiguous positive and the outcome of this interactions might be due to a balance between these multi-directional processes. In summary, there are more publications on the inducer role of infections in CD, and the few ones advocating the protective role should be further explored. The present review expend on several avenues that can be studied to understand the protective cross-talks between infectious agents and CD. Apprehending them can potentially suggest new therapeutic strategies for CD.

## Author contributions

AL: designed, wrote, edited, submitted, MA: designed, wrote, AS: literature search, revised, reviewed, TM: literature search, edited, and revised.

### Conflict of interest statement

The authors declare that the research was conducted in the absence of any commercial or financial relationships that could be construed as a potential conflict of interest.
